# Physicochemical and Microbiological Qualities’ Assessment of Popular Bangladeshi Mango Fruit Juice

**DOI:** 10.2174/1874285801812010135

**Published:** 2018-04-30

**Authors:** Ruhul Amin, Shafkat S. Rahman, Mahboob Hossain, Naiyyum Choudhury

**Affiliations:** 1 Biotechnology Program, Department of Mathematics and Natural Sciences, BRAC University, Dhaka-1212, Bangladesh; 2 Perfetti Van Melle Bangladesh Pvt. Limited, Mouza# 7 NO, Kewa (Beraider Chala), Gilar Chala Rd, Vill# Beraider Chala, P.O.# Gilaberaid, P.S.# Sreepur, Gazipur-1740, Bangladesh; 3 United Surgical (BD) Ltd, Reliance Industrial Park, Plot# 659-661, Islampur, Kadda, Gazipur-1702, Bangladesh; 4 Bangladesh Atomic Energy Regulatory Authority (BAERA), E-12/A, Shahid Shahabuddin Shorok, Dhaka-1207, Bangladesh; 5 Microbiology Program, Department of Mathematics and Natural Sciences, BRAC University, Dhaka-1212, Bangladesh

**Keywords:** Mango juice, Microbial, Nutrition quality, Monosaccharide, Carbohydrate profile, Fecal coliform

## Abstract

**Introduction:**

Mango juice has always been considered as a delicious, nutritious popular drink, but processed juice may not always be safe due to chemical and microbial risks. Determination of physicochemical and microbiological qualities of some packed mango juices of Bangladesh will help consumers to know the present scenario.

**Material and Methods:**

Six commercially available different juice samples were collected from the market. Carbohydrate profiles were determined using HPLC, crude protein content was calculated using the Kjeldahl method and other parameters were determined by standard AOAC methods. Standard culture techniques were followed to assess the total viable count (TVC), *E. coli* and other fecal coliforms.

**Results:**

The highest quantity of monosaccharide (58.88%) was recorded in the AC1ME5 brand, while the lowest in Homemade (5.648%) and MN1GL2 (9.867%). The maximum content of acidity recorded was 0.24% and minimum 0.21%. The TSS content of all samples varied from 19% to 12%. The highest quantity 6.87% and the lowest 3.62% of reducing sugar were recorded. Most of the mango juices were low in protein and very low/negligible in fat content. Total viable count of different types of fruit juices varied from 1×10^3^ - 3×10^3^ CFU/ml. No significant amount of *E. coli* and fecal coliform was detected.

**Conclusion:**

It can be concluded that the locally available mango juices contain a safe level of nutritional and microbial elements for human consumption, but not highly satisfactory.

## 1 INTRODUCTION

Among many local fruits, mango is the most popular in Bangladesh because of its taste and nutritional value. Fresh mango is available in a particular season. Due to popularity and consumer demand, many fruit juice companies have developed to capitalize on emerging market [1]. Mango juice is considered as the most preferred non-alcoholic beverage worldwide to all age groups [2]. Each cup of mango juice adds both vitamins A and C to diet and contains about 30 grams of sugary carbohydrates (sugars – glucose, fructose, maltose, sucrose; dietary fibers) [3]. Although moderate to high level of carbohydrates causes little fluctuation in blood-sugar level, this process makes the body maintain appetite through an easier metabolism. Non-fat originated calories, make the juice easily digestible, an energy providing drink. The important nutrient from mango juice helps to maintain healthy eye function and growth, healthy skin tissue and gene transcription. Hartwell claims in his book “Plants Against Cancer,” that the phenols in mangoes, such as quercetin, isoquercitrin, astragalin, fisetin, gallic acid and methyl gallate, as well as the abundant enzymes, have healing and anti-cancer capacities. In gallbladder cancer, a protective effect of mango consumption had been proven [4]. It meets the vitamin and energy requirements of children of 6-24 months of age at three servings a day and at the FAO average breastfeeding frequency [5]. Mangoes also contain a lot of tryptophan, the precursor of the “happiness hormone” serotonin [6]. Mango juice also is a good source of calcium and iron. Iron helps the body to eliminate free radicals, while calcium assists in the formation of healthy teeth and bones. Phytochemicals and antioxidants [7] in mango juice are beneficial to prevent many diseases along with cancer. A study in New Zealand aimed to determine the reason for lower cancer rate in native Maori population compared to European descendants. The Maori eat 25 foods in greater quantities, five of them showed strong anti-cancer effects. Those are carotenoids and flavonoids containing, watercress, papaya, taro leaves, green banana and mango [8]. Mango juice also provides protection against arteriosclerosis.

Processed mango juice may not always be safe due to chemicals (additives as sodium benzoate, sulfur dioxide) used in ingredients and the heavy load of microbes (T.P.C., yeast, mold). Sometimes producers do not maintain proper quality parameters as pH, acidity, total soluble solids and aseptic condition. For these, the product failed to maintain the nutrition value and taste or deliciousness. Producers apply chemical preservatives that can inhibit all types of microbial growth [9]. Commonly used processed fruit juice preservatives such as sulfur dioxide (SO_2_) and sodium benzoate are detrimental to health and significantly damage the vegetative cells [10]. The sulfites inhibit yeasts, molds and bacteria are the most effective as inhibitors of browning in foods. They also reduce the microbial growth and increase the shelf life of juice products [2]. Refrigeration and sterilization [11] are popular methods for the preservation of mango juice used to destroy pathogenic microorganisms and to preserve the color, aroma and chemical quality. The major ingredients of juice are water [12], sugar, glucose, natural fruit pulp, sodium CMC and may also carry microbial contaminants. Food-borne illness is commonly caused by some chemicals and certain bacteria or their toxins. The most common food-borne pathogenic bacteria, associated with mango juice, are *Bacillus cereus, Escherichia coli, Salmonella* spp., *Staphylococcus aureus* etc [2]. In pregnant women, a heavily infected fetus may lead to spontaneous abortion, stillbirths, or sepsis in infancy. Contaminated juice with pathogens as *E. coli* and *Salmonella* spp. has caused numerous ailments and reported fatalities [13].

For 4000 years, mango has been cultivated in the sub-continent [14]. The grafted mango plants are concentrated in a few places in the North-Western region and unknown varieties (seedling mangoes) are grown in other parts of Bangladesh [15]. The Rajshahi zone under mango cultivation during 2003-04 was about 50991 hectares with a total production of about 242605 metric tons [16]. North-Western district Chapai Nawabganj alone with around 50,000 mango groves produced a total of 172 thousand tons of mango in 2010 [17]. In Bangladesh, 90% of the existing mango plants are raised from seeds [18] and for the lack of suitable variety, Bangladesh now is in a decreasing trend in terms of production [19].

The consumption of fruit juice continues to increase in Bangladesh because of taste, nutrition value and lifestyle demand. So, from the public health point of view, it is quite important to know the physicochemical and microbiological quality of the increasingly popular fruit juices available in the market. Determination of these parameters of some commercially packed mango juices of Bangladesh will help consumers to know the present scenario or the condition of Bangladeshi processed juice.

## 2 MATERIAL AND METHODS

### 2.1 Sample collection

Six samples, which are most popular and available all over the country, of commercially packed juices [*FR1TK4 (Mango Fruit Drinks) 250 ml, FR1TO6 (Mango Fruit Drinks) 250 ml, MN1GL2 (Mango Juice) 250 ml, ST1RP1 (Fruit Juice) 200 ml, SH1ZC3 (Mango Drinks) 250 ml, AC1ME5 Mango Drinks], were collected from the market of Dhaka (Mohakhali area) in January 2014. One juice was prepared as standard (Homemade) from mango pulp, water and sugar.

### 2.2 Physicochemical Test Method

All seven samples were collected in sterile containers, kept in the icebox, maintained at 4ºC during transportation to the BRAC University laboratory and analyzed within 2-3 hours. The pH value was measured with a pH meter (Mettler Toledo, Switzerland).

#### 2.2.1 Total soluble solids

Total soluble solids, primarily sucrose, fructose and glucose, were measured. Citric acid and minerals in the juice also contributed to the soluble solids. Brix is reported as “degrees Brix” and is equivalent to a percentage. For example, a juice measured as 12 degrees Brix has 12% total soluble solids. The national standards state that the minimum Brix sugar/ acid ratio for navel oranges is 10:1 [20-22].

#### 2.2.2 Determining Acidity

Citric acid and a small amount of malic and tartaric acid were added in juice for its tartness and unique taste. The amount of acid present in the juice was reported as the percent citric acid. A titration with sodium hydroxide was used to calculate the value (Appendix A) [21, 23].

#### 2.2.3 Kjeldahl Method to Determine Protein

Juice was digested with a strong acid (H_2_SO_4_) so that it released nitrogen which could be determined by a suitable titration technique. The amount of protein present was calculated from the nitrogen concentration. A conversion factor of 6.25 (equivalent to 0.16 g nitrogen per gram of protein) was used for applications. This was only an average value and each protein has a different conversion factor depending on its amino-acid composition. The Kjeldahl method can conveniently be divided into three steps: digestion, neutralization and titration. Anhydrous sodium sulfate and a catalyst (copper) were introduced to alleviate the boiling point of the medium (from 337°C to 373°C). The initially very dark-colored medium had become clear and colorless to indicate that the chemical degradation of the sample was completed [24, 25].

#### 2.2.4 Fat Content Determination

The fat content of the sample was determined as free and total fat [26]. Free fat was extracted from the lyophilized sample by Soxhlet using ether as a solvent. The total fat content was determined by the acid-hydrolysis method [27]. Samples (1.5 g) were digested with dilute hydrochloric acid (5 ml) for about 45 minutes in a water bath. The mixture obtained was then extracted with a combination of solvents comprising of methanol (2.5 ml), diethyl ether (7.5 ml) and petroleum ether (7.5 ml). Thereafter, the mixture was centrifuged, ether–fat layer was decanted and evaporated and the fat content was measured (Appendix B) [25].

#### 2.2.5 Total Reducing Sugar (TRS)

A number of chemical methods were used to determine monosaccharides and oligosaccharides which are based on the fact that many of these substances are reducing agents. Those reducing agents can react with other components to yield precipitates or colored complexes which could be quantified. Acidity was measured according to the AOAC method [27] and expressed in g/L as citric acid (Appendix C).

#### 2.2.6 Carbohydrates Profile Chromatographic Methods

HPLC is commonly used to separate and identify carbohydrates [28] because of rapid, specific, sensitive and precise measurements. Sugar content tests were performed using high-pressure liquid chromatography [HPLC Model# CTO20A, Shimadzu, Japan]. The solution (2% w/v) was prepared by the doubled refined distilled and sonicated (10 minutes) water. The sample was injected into the 20 μl column (solid phase). The separation was conducted at 80°C with the mobile phase water at 1.3 ml/min flow rate. The identification of monosaccharide was done by comparing the retention times of individual sugars in the reference vs. standard solution. The quantitative assays were made of the carbohydrates as fructose, glucose, sucrose, maltose, malt triose and maltotetraose. The contents of those compounds were assayed based on the comparing peak areas obtained in the examined samples with those from the standard. To make the presentation of the obtained results more comprehensive, the following was calculated: total sugars, fructose to glucose ratio and total monosaccharides. The calculation was done by the Post Run software (Lab Solution).

### 2.3 Microbiological Test Method

The standard test procedure was followed [29] and appropriate selective media were inoculated for microbiological analysis for the quantitative determination of total viable count (TVC), fecal coliform, *E. coli* [30].

#### 2.3.1 Total Viable Count

NaCl (0.9 gm) was diluted in 10 ml of water for NaCl–0.9% saline solution. 1:10^-3^ dilutions were prepared by aseptically transferring the well-mixed sample to the desired volume of diluent. 2.8 gm of dehydrated Nutrient Agar medium (Brand: Sigma) was taken in 100 ml of deionized or distilled water. Flask was placed in a slowly heated water bath to reach 90°C. 1 ml of rosolic acid solution was added per 100 ml of medium with a pipette. The medium was cooled down to about 45-50°C. Then ~200 µl sample was spread on selective solidified Petri dishes aseptically and incubated for 24 hours at 37±0.5°C.

#### 2.3.2 Confirmation of E. Coli Using MacConkey Agar Media

Dehydrated MacConkey Agar medium (5.15 gm) was taken in 100 ml of deionized or distilled water. Flask was placed in a heated water bath to reach 90°C. 1 ml rosolic acid solution was added per 100 ml of medium. The medium was cooled down to about 45-50°C. Then ~200 µl sample was spread on selective Petri dishes and incubated for 24 hours at 37±0.5°C. A colony of each plate of the same dilution was counted and confirmed that selected plates were containing not more than 100 CFU per petri dish. Counted colony for each dilution was averaged and multiplied by the dilution factor. *E. coli* was differentiated from other coliforms also by growing in EMB agar media.

#### 2.3.3 Fecal Coliform Test

Dehydrated MFC medium (5.210 gm) was taken in 100 ml of deionized or distilled water. Flask was placed in a slow heated water bath at 90°C. One milliliter of rosolic acid solution was added per 100 ml of the medium. The medium was cooled down to about 45-50°C. Then ~200 µl sample was spread on selective Petri dishes and incubated for 22±2 hours at 44.5°C [31].

## 3 RESULT

### 3.1 pH of Various Juice Samples

Fruit juices have a low pH because they are comparatively rich in organic acid. The overall range of pH is 2 to 5 for common fruits with the most frequent figures being between 3 and 4. In this study pH of the fruit juices varied from 3.55 to 3.80 as shown in Table **1**. The highest pH (3.80) was found in ST1RP1, followed by MN1GL2, Homemade juice, SH1ZC3, AC1ME5, FR1TK4 and FR1TO6.

### 3.2 Total Soluble Solids of Collected Juice Samples

The TSS content is significantly influenced by the percentage of solid materials (mango pulp, sugar, glucose and other ingredients) dissolved in water in the juice. Sometimes, the producer adds a sweetening agent instead of sugar and glucose. On the other side, some producers add other ingredients as sodium CMC to increase TSS artificially. TSS value of the homemade juice was 19% which was the highest. TSS of FR1TK4 was 12.8%, FR1TO6 12%, MN1GL2 13.5%, ST1RP1 12.75%, SH1ZC3 12.25% and 12% recorded in AC1ME5 juice as shown in Table (**1**) , Fig. (**1**).

### 3.3 Total Acidity of the Collected Juice Samples

The total acidity of fruit juices is due to the presence of a mixture of organic acids, whose composition varies depending on the fruit nature and maturity of the pulp or ingredients used which were added during juice processing. Organic acids take the lead in importance for characteristics and nutritive value of fruit juices and confer individual originality among natural beverages. Acidity (as citric acid) varied significantly in different types of fruit juices as shown in Table **1**. Acidity (as citric acid) of homemade juice was 0.21%, FR1TK4 0.23%, FR1TO6 0.24%, MN1GL2 0.21%, ST1RP1 0.21%, SH1ZC3 0.23% and AC1ME5 juice contained 0.21% acidity.

### 3.4 Protein Content in Collected Juice Samples

Most of the fruit juices were low in protein content. Protein is insoluble in fruit juice so a considerable proportion of the protein content is present in the fruit juices. Homemade juice contained only 0.1%, FR1TK4 0.11%, FR1TO6 0.18%, ST1RP1 0.12%, AC1ME5, MN1GL2 and SH1ZC3 contained 0% protein as displayed in Table (**1**).

### 3.5 Fat Content in Collected Juice Samples

Fruit juice does not contain fat; somehow a little amount could be present in raw ingredients. Fat was not detected in homemade juice, FR1TK4, ST1RP1 and SH1ZC3 samples. FR1TO6 contained 0.17%, MN1GL2 had 0.13%, AC1ME5 juice contained only 0.11% as shown in Table (**1**).

### 3.6 Total Reducing Sugar of Collected Juice Samples

It is estimated that reducing sugar and total sugar content increased with the advanced ripening of fruits. The combined effect of the stages of maturity and ripening conditions significantly affected the reducing sugar and total sugar content of the fruit pulp and juices. Homemade juice contained 3.76%, FR1TK4 5.85%, FR1TO6 6.87%, MN1GL2 3.6%, ST1RP1 4.39%, SH1ZC3 6.8% and AC1ME5 juice contained 5.3% TRS. The chart is presented below in Table (**1**).

### 3.7 Carbohydrate Profile of Collected Juice Samples

**Fig. (2):**
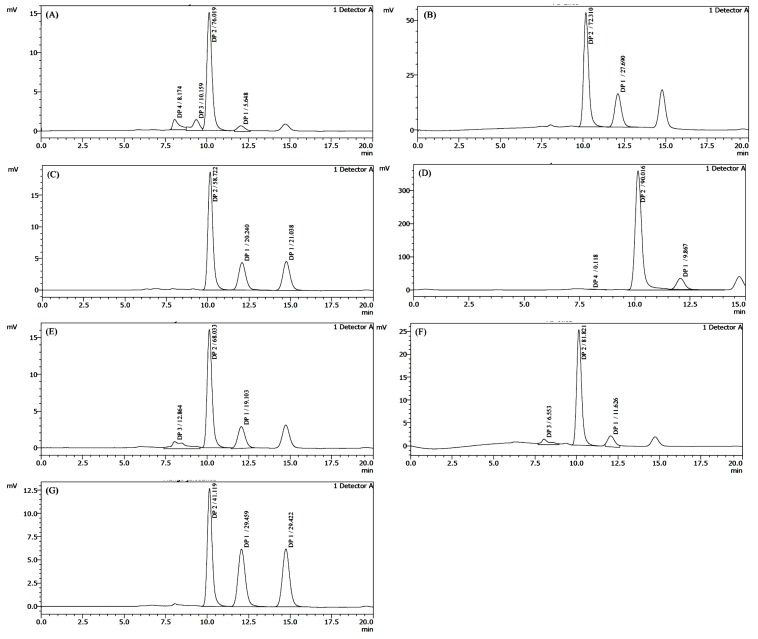
HPLC graph showing carbohydrate profiles of - (A) Homemade juice, (B) FR1TK4, (C) FR1TO6, (D) MN1GL2, (E) ST1RP1, (F) SH1ZC3 and (G) AC1ME5 brands. DP - Dextrose Profiles; DP1 - Glucose or fructose, DP2 - Sucrose, DP3 - Triose, DP 4 – Tetraose.

In this study, Carbohydrate Profile was analyzed by the HPLC and dextrose profile was segregated from other oligosaccharides. Homemade juice contained 5.648% monosaccharides which was a very low percentage and it came only from mango pulp. FR1TK4 contained 27.69%, FR1TO6 41.27%. MN1GL2 9.867% and AC1ME5 contained 58.88% monosaccharides. SH1ZC3 11.626% and ST1RP1 contained 19.103% monosaccharides. The below (Table **2** and Fig. **2**) show the compression of Dextrose profile between the all mango juice samples.

### 3.8 Total Viable Count of Collected Juice Samples

Microbial count of different fruit juices is shown in Table **3**. From the results, it is apparent that total viable count (microbial load) showed the presence of bacteria in the range of 1×10^3^ - 3×10^3^ CFU/ml maximum, which is lower than the Gulf standard [32] for foods. From the table, it can be found that FR1TK4 3×10^3^ CFU/ml as shown in Fig. (**3A**), FR1TO6 1×10^3^ CFU/ml, MN1GL2 2×10^3^ CFU/ml and SH1ZC3 contained 3×10^3^ CFU/ml respectively as shown in Fig. (**3B**). There was no viable count recorded in the sample homemade mango juice, ST1RP1 and AC1ME5 brand.

### 3.9 *E. coli* and Fecal Coliform Count in Collected Juice Samples

*E. coli* was present only in one sample (FR1TO6) containing 1×10^3^ CFU/ml and was absent in MN1GL2, FR1TK4, SH1ZC3, homemade mango juice, ST1RP1 and AC1ME5 mango drinks pack. No other fecal coliform was present in any samples.

## 4 DISCUSSION

Though there were many different fruit drinks available in Bangladesh market and their test, quality and nutrition properties are also different. For the limitation of time, equipment and laboratory facilities, only mango juice was taken into consideration for this study.

Fruit juices have a low pH because they are comparatively rich in organic acid. As per the observation of Saeed *et al*. [33], the pH of Sample I to VI was 4.21 to 4.6 and Cadena *et al*. [34] reported 4.10 to 4.13 pH, while others recorded pH 3.95 [23] and 3.79 [35] in their observation. In this study, the pH of the fruit juices varied from 3.55 to 3.80 as shown in Table **1**. The highest pH 3.8 was shown in ST1RP1 and the levels of all samples (3.55 to 3.8) were within the limits of BSTI standard for fruit juice [20, 36].

There is a notable disparity between the result of this study and the results reported by Saeed *et al*. [33], especially for TSS (Total soluble solid), one of the most important parameters of the investigation. As per the observation of that study, six samples contained 5.1%, 9.8%, 6.5%, 5.1%, 12.9% and 10.3% of TSS. Cadena *et al*. [34] observed 7.5%, 14.0%, 7.83%, 7.5%, 7.5% and 7.33% in six samples, while others reported 8.14 to 11.91 [23, 35]. As per the observation of those studies, there was unequal variance in the juice sample. The lowest TSS is 5.1 and the highest is 14.0 whereas, in the study, the lowest was found to be 12.0 and the highest was found to be 19.0. So, the juice samples used in this study were maintained average TSS which was within the limit of Bangladesh regulatory authority (BSTI). The TSS content in MN1GL2 and homemade juice was higher than that of other juices as shown in Fig. (**1**).

The total acidity of fruit juices was due to the presence of a mixture of organic acids, whose composition varies depending on the fruit’s nature and maturity of the pulp or ingredients used that were added during processing. Organic acids took the lead in importance for the characteristics and nutritive value of fruit juices and deliberated individual originality among beverages. For acidity parameter observed by Saeed *et al*. [33] and Cadena *et al*. [34]; six samples contained 0.098% to 0.259% and 0.1479% to 0.1565% respectively. 0.18% and 0.34% were also reported [23, 35]. In the present study, there was no significant variation in the total titrable acidity between samples as shown in Table **1**. The maximum content of total titrable acidity (0.24%) was recorded for FR1TO6 while it was minimum (0.21%) in MN1GL2 and AC1ME5 drinks. Many producers added ascorbic acid to their products to make up for acidity; this could be the cause for the similar content of acid (0.21% to 0.24%) in mango juices in this study. The result of this study was congruent with others.

Most of the common juices were found to have low protein level. In this study, protein content (0.8%) in FR1TO6 was comparatively higher than in other juices as shown in Table **1**. Saeed *et al*. [33] observed that Sample I to VI contained 0.175%, 0.175%, 0.145%, 0.016%, 0.133% and 0.179% protein respectively. In this study, percentage comparison was similar or slightly lower than that study. Homemade juice contained only 0.1% protein, FR1TK4 0.11%, FR1TO6 0.18%, ST1RP1 0.12% and MN1GL2, SH1ZC3 or AC1ME5 juice contained no protein content at all.

Low amount of fat detected in FR1TO6 (0.17), MN1GL2 (0.13) and AC1ME5 (0.11), but other four samples including Homemade juice contained no fatty substance as shown in Table **1**. The source of fat remained ambiguous.

The variations in reducing sugar of the samples may be attributed to the formulation of the different manufacturer and combined effect of maturity stages and ripening conditions significantly affected the reducing sugar and total sugar content of the fruit pulp and juices. As per Table **1**, homemade juice contained 3.76% TRS, FR1TK4 5.85%, highest 6.87% FR1TO6, lowest 3.6% MN1GL2, ST1RP1 4.39%, SH1ZC3 6.8% and AC1ME5 juice contained 5.3% TRS. As per the observation of Tasnim *et al*. [37], the quantity of reducing sugar varied between 3.37% to 9.99% in mango juices.

Commonly occurring monosaccharides in foods are glucose, fructose, galactose, arabinose and xylose. The reactive centers of monosaccharides are the carbonyl and hydroxyl groups [38]. In the present study, carbohydrate profile was analyzed by the HPLC and dextrose profile was segregated from other oligosaccharides. Homemade juice contained 5.648% monosaccharides which is a very low amount and it came only from mango pulp because only this component was used in the preparation of the homemade sample without adding glucose or fructose or any invert sugar. So, it can be stated that the average 5% monosaccharides come from mango pulp. So, those juices containing more monosaccharide indicate that they were produced with more mango pulp or more glucose-fructose was externally added. Dextrose profile 1 (DP 1) indicates monosaccharide in the graph of HPLC generated result shown in Fig. (**2**). The juices which contained high quantity of DP 1 are consumer preferred due to instant energy. But, high amount of fructose in processed food is also not salubrious [39, 40]. A correlation between flavor and sugar concentration was also reported [41]. FR1TK4 27.69%. FR1TO6 41.27%. MN1GL2 9.867%, AC1ME5 58.88%. SH1ZC3 11.626% and ST1RP1 contained 19.103% monosaccharides as shown in Table **2**. Among those, fructose was detected in FR1TO6 and AC1ME5.

**Fig. (3):**
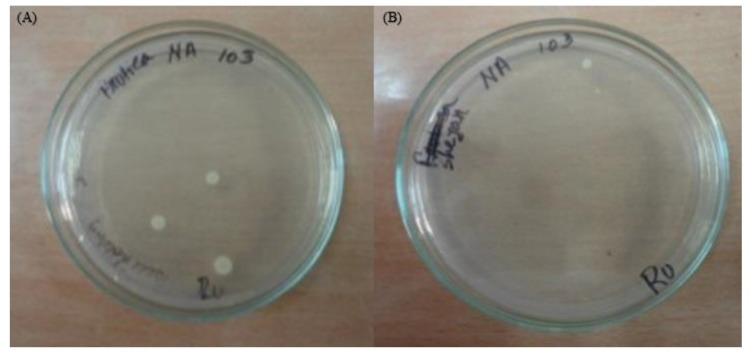
Growth on Nutrient Agar media at 37°C of (A) FR1TK4 and (B) SH1ZC3 mango juice.

Plant foods, especially plant juices, tend to have redox potential (Eh) values from 300 to 400 millivolts. Having such a high redox potential is an indication of the availability of a sufficient amount of free oxygen accessible to aerobic microbes. Thus, the survival and growth of aerobic bacteria and molds in such products are high and with the same microbial groups being responsible for decomposition of the same products [42]. Lack of hygiene, production tardiness and contaminated ingredients were major detrimental factors for the quality of fruits juice [43]. The range of microbial counts (1×10^3^ to 3×10^3^ CFU/ml) recorded in the fruit juices analyzed in this study was relatively lower than the microbial load reported in some earlier works (Table **3** , Figs. **3A** and **3B**).

As per the study of Tasmina *et al*. [2] TVC 2.0x10^2^ to 3.2x10^2^ CFU/ml was found in different types and brands of mango juice samples. The study of Tsige Ketema [44] found 6.2x10^3^ - 3.1x10^7^ CFU/ml TVC and other microbes were higher than the result of this study. However, the recommended specifications for fruit juices served in the Gulf region suggest that the maximum count permitted for total colony count of coliforms, yeast and molds is 1x10^4^, 100, and 1x10^3^ CFU/ml, respectively. On the basis of Gulf standards [32], it is clear that the colony counts of almost all the microbial groups in fruit juices collected for this study have not exceeded the standard. In this study, TVC of different samples varied from 1×10^3^ to 3×10^3^ CFU/ml. No other *Fecal coliform* was detected in these juices, except *E. Coli* was detected only in one sample as 1×10^3^ CFU/ml. Commercially packed juices are far less contaminated than those sold by street-vendors [45]. The processing equipment of the juices may contribute to the number of bacterial and fungal species. We know that chemical preservatives significantly decreased the microbial load in fruit juices [35]. Regulating the microbial safety of facilities to be used for processing and the use of good quality fruits and surface disinfection besides cleaning with pure water could certainly ameliorate the microbiological quality of the juices [46]. To ensure longer shelf-life and safety of the juices against fungi and molds, producers generally use approved food additives. Many organic acids with Generally Regarded as Safe (GRAS) status have been currently used for the preservation of many foods and juices. However, these low counts may not necessarily pose a hazard to the health of consumers [47].

From the study, it can be concluded that physicochemical and microbial assessment of ST1RP1, MN1GL2, SH1ZC3, FR1TK4 and AC1ME5, divulged that the quality was lower than the homemade standard. The overall result of FR1TO6 was very poor and showed the highest deviation from standard in every parameter. This study did not provide any information regarding food additives, which might have been used in the samples, but erected a pathway towards advanced studies.

## CONCLUSION

This work has shown that the locally available mango juices contain the safe level of nutritional and microbial elements for human consumption, but the overall quality is not very good. From the data presented in the current study, it can be concluded that the nutritional quality has a variance among brands. The basic quality parameters such as pH, TSS, fat content, protein content, acidity, acid value and carbohydrate profile were maintained within the limit of BSTI. The microbial growth was found to be less frequent among some juice samples, but, all of them were not free from microbial load. The samples contained TVC because of lack of monitoring and maintaining GMP (Good Manufacturing Practice). Most of the Bangladeshi companies use preservative treated pulp in a hot filling unit or an aseptic filling system. But there are many other technologies as Hyperbaric Pressure, Hyperbaric + CO_2_, Pulsed electric field (PEF), Ultrasonic Membrane, Pulsed light, Magnetic field, Irradiation [48] which can replace the old technology. The Government-authorized institute as BSTI should undertake preventive investigations to check the microbial and chemical quality of the fruit juices. The government authorized department should also take initiatives for providing training to the technical staff to increase producers’ awareness on maintaining the rules and regulation of GMP, HACCP and FSSC for production. They can take initiative to increase awareness among consumers for pre-checking the batch manufacturing date.

## Figures and Tables

**Fig. (1):**
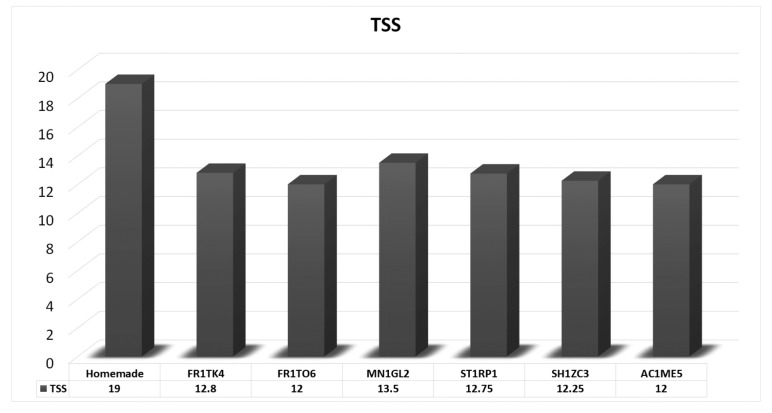
Total soluble solids of collected juice samples.

**Table 1:** Physicochemical parameters of collected juice samples.

** # **	**Name of Juice**	**pH**	**TSS (%)**	**Acidity (%)(as citric acid)**	**Protein (%)**	**Fat (%)**	**TRS (%)**
1	Homemade juice	3.77	19	0.21	0.10	0	3.76
2	FR1TK4	3.63	12.8	0.23	0.11	0	5.86
3	FR1TO6	3.55	12	0.24	0.18	0.17	6.87
4	MN1GL2	3.76	13.5	0.21	0	0.13	3.6
5	ST1RP1	3.8	12.75	0.21	0.12	0	4.39
6	SH1ZC3	3.73	12.75	0.23	0	0	6.8
7	AC1ME5	3.65	12	0.21	0	0.11	5.2

**Table 2:** Carbohydrate Profile of collected juice samples.

** # **	**Name of Juice**	**Dextrose (%)**	**Sucrose (%)**	**Triose (%)**	**Tetraose (%)**
1	Homemade juice	5.648	76.019	10.159	8.174
2	FR1TK4	27.69	72.31	0	0
3	FR1TO6	41278 (20.24 + 21.038)	58.722	0	0
4	MN1GL2	9.867	90.016	0	0.118
5	ST1RP1	19.103	68.033	12.864	0
6	SH1ZC3	11.626	81.821	6.553	0
7	AC1ME5	58.881 (29.459 + 29.422)	41.119	0	0

**Table 3:** Total viable count of collected juice samples.

** # **	**Name of Juice**	**TVC (CFU/ml)**
1	Homemade juice	0
2	FR1TK4	3.0×10^3^
3	FR1TO6	1×0^3^
4	MN1GL2	2.0×10^3^
5	ST1RP1	0
6	SH1ZC3	3.0×10^3^
7	AC1ME5	0

## LIST OF ABBREVIATIONS

HPLC: = High Performance Liquid Chromatography
AOAC: = Association of Official Agricultural Chemists
TVC: = Total viable count;
pH: = Negative logarithm of hydrogen ion concentration
TSS: = Total soluble solids
*E. coli*: = *Escherichia coli*
FAO: = Food and Agriculture Organization
SO_2_: = Sulfur dioxide
CMC: = Carboxymethyl cellulose
spp.: = Species
TRS: = Total Reducing Sugar
SSC: = Soluble Solid Content
μl: = Microliter
°C: = Degree Celsius
ml/min: = Milliliter per minute
vs: = Versus
EEC: = European Economic Community
FDA: = Food and Drug Administration, United States Public Health Service
h: = Hour
MFC: = Membrane Fecal Coliform
mm: = Millimeter
N: = normal
NaOH: = Sodium hydroxide
L: = Liter
UV: = Ultraviolet
CFU: = colony-forming unit
CH_3_CH_2_OH: = Ethanol
C_6_H_12_O_6_: = Glucose
DP: = Dextrose profile
H_2_O: = Water
H_2_SO_4_: = Sulfuric Acid
TFC: = Total Fecal coliform count
w/v, v/v: = Weight per Volume, volume per volume
(NH_4_)2SO_4_: = Ammonium sulfate
Wt, ml, kg, g, gm: = Weight, Milliliter, Kilogram, Gram
*et al*.: = Associates
US$: = United States dollar
MT: = Metric ton
GRAS: = Generally Regarded as Safe
BSTI: = Bangladesh Standards and Testing Institution
GMP: = Good Manufacturing Practice
CO_2_: = Carbon dioxide
PEF: = Pulsed electric field
HACCP: = Hazard analysis and critical control points
FSSC: = Food safety system certification
Conc.: = Concentration.

## APPENDIX A

### Acidity (Citric acid content) determining calculations

$\mathrm{Titrable}\;\mathrm{acidity}\;(\%)\;=\;\frac{T.V\times \mathrm{Factor}\text{∗}}{W}$

Where TV = Titer value of the sample in ml.; W = quantity of the sample taken for the test in ml.

Factor for - Citric acid: 0.0064 (Citrus fruit), Malic acid: 0.0067, Tartaric acid: 0.0075

## APPENDIX B

### Fat Content Determination

% of Reducing Sugars = F × 200 × 100/(V1 × W)

Where (Net ml KBrO_3_-KBr) = (ml KBrO_3_-KBr for Sample – ml KBrO_3_-KBr for Blank)

For 25 ml juice sample titrated with 0.0247N KBr_3_-KBr

## APPENDIX C

### Total reducing sugar (TRS) determining calculations

% of Reducing Sugars = F x 200 x 100/ (V1 x W)

Where F = Factor for 5 ml of Fehling’s Solution; V1 = Volume of sample solution consumed; W = Sample weight in gm
